# Preliminary Outcomes of a Digital Remote Care Solution for Colorectal Cancer Patients

**DOI:** 10.3390/cancers17162622

**Published:** 2025-08-11

**Authors:** Marta Chaparro-Mirete, Cristina González Callejas, María de los Ángeles García-Martínez, Jorge Ramos-Sanfiel, Maria Sol Zurita-Saavedra, Paola De Castro-Monedero, Javier Gómez-Sánchez, Ángela Argote-Camacho, Alfredo Ubiña-Martínez, Cristina González-Puga, Carlos Garde-Lecumberri, Teresa Nestares, Benito Mirón-Pozo

**Affiliations:** 1 General and Digestive Surgery, San Cecilio Clinical University Hospital, 18007 Granada, Spain; mchaparrom.md@gmail.com (M.C.-M.); benimp75@gmail.com (B.M.-P.); 2 Colorectal Surgery Unit, General and Digestive Surgery Service, San Cecilio Clinical University Hospital, 18007 Granada, Spain; callejascris21@gmail.com (C.G.C.); mariadelosangeles_garcia@hotmail.com (M.d.l.Á.G.-M.); jorgers91@gmail.com (J.R.-S.); marisolzuritasaavedra@gmail.com (M.S.Z.-S.); decastromonederopaola@gmail.com (P.D.C.-M.); javiergomezsanchez.jg@gmail.com (J.G.-S.); angelitaxa@hotmail.com (Á.A.-C.); fredyubimar@gmail.com (A.U.-M.); crisgona2@hotmail.com (C.G.-P.); carlos.garde.sspa@juntadeandalucia.es (C.G.-L.); 3 Department of Physiology, Institute of Nutrition and Food Technology “José Mataix”, Center for Biomedical Research, University of Granada, 18016 Granada, Spain

**Keywords:** colorectal cancer, remote monitoring, quality of life, mobile applications: digital health, patient compliance, multimodal therapy

## Abstract

**Background/Objectives**: Colorectal cancer (CRC) ranks third in the Western world in cancer incidence and second as the cause of cancer-related deaths. Despite advances in perioperative care, minimizing postoperative morbidity is crucial in clinical practice. Digitalization of the healthcare process plays a key role in genuinely and effectively engaging patients. Our aim was to evaluate a digital solution for remote monitoring of patients with CRC, from surgery indication to postoperative discharge. **Methods**: We developed a digital solution using Value Stream Mapping (VSM) to identify patient care flow and Lean Sigma for optimization and efficiency. We incorporated the Enhanced Recovery After Surgery (ERAS)/RICA pentamodal recommendations to create a program with an individualized schedule for each patient, who received tailored educational, medical, and practical information at every stage of the process. **Results**: A total of 193 patients used the digital solution, with >75% adhering to ERAS recommendations. The median length of hospital stay was 5 days, with low adherence leading to 3.4 (*p* = 0.628) or 3.27 (*p* = 0.642) extra days in the hospital compared to patients with intermediate and high adherence, respectively. The mean comprehensive complication index (CCI) was 9.1/100, which was higher in patients with low adherence (15) versus intermediate (8.17; *p* = 0.027) and high (7.42; *p* = 0.011) adherence. An increase in self-perception of quality of life by 9.2% was identified at the end of the process compared to the outcome at the beginning (*p* = 0.09), and 80% rated their overall satisfaction with the care process as 8 or higher out of 10. **Conclusions**: The digital solution facilitates the monitoring of CRC care and implementation and adherence to ERAS recommendations, improving patient engagement and satisfaction.

## Simple Summary

Despite the significant technological advances in recent decades, the integration of digital solutions into routine clinical practice remains limited. In colorectal cancer (CRC), Enhanced Recovery After Surgery (ERAS) protocols have been shown to improve postoperative outcomes, yet consistent implementation and monitoring remain a challenge. Recent value-based healthcare (VBHC) models highlight the importance of incorporating patients’ perspectives and experiences into care delivery. We developed a digital platform for the remote monitoring of patients with CRC to promote active participation in their care, provide personalized follow-up, monitor adherence to ERAS protocols, and evaluate both postoperative complications and patient-reported outcomes. Preliminary results from its implementation suggest an association between ERAS protocol adherence, length of hospital stay, and postoperative recovery. These preliminary findings suggest that digital health solutions have the potential to facilitate more personalized, coordinated, and patient-centered care models.

## 1. Introduction

Colorectal cancer (CRC) is the third most common cancer in the Western world and the second leading cause of cancer-related death. More than 9 million CRC surgeries are performed each year, with an estimated increase of over 40% expected in the coming decades. The complications associated with both CRC and its surgical treatment significantly impact patients and healthcare systems [1].

Despite advancements in perioperative care, postoperative-related morbidity remains a challenge for the health system, ranging between 20% and 37% according to various studies. Improving the recovery process for patients whose quality of life deteriorates in the medium to long term [2,3], the morbidity associated with CRC surgery, and the perception of the entire process that patients go through are fundamental goals in clinical practice [4].

In the mid-1990s, the Danish surgeon Henrik Kehlet proposed a multidisciplinary approach that evolved into the concept of Enhanced Recovery After Surgery (ERAS). It establishes the functional capacity as a key factor in achieving a favorable postoperative outcome. It focuses on multimodal and patient-centered management to improve the functional capacity and optimize the outcome after surgery [5,6,7].

The ERAS protocol is built upon several pillars: 1. Prehabilitation, which involves preparation, information, and follow-up prior to surgery to optimize the patient’s baseline condition; 2. Perioperative care, including minimally invasive surgery, control of opioid and fluid management, early oral nutrition, and mobilization; 3. Transversal and multimodal care, conducted by an expert team of surgeons, anesthesiologists, and nurses; and 4. Close postoperative follow-up both during the hospital stay and after discharge [8,9,10,11,12,13].

The number of trials suggesting that prehabilitation may lead to a clinically significant improvements in patients’ preoperative functional status and surgical outcomes has increased in recent years, with their level of evidence and strength of recommendation increasing [14]. A recent multicenter randomized clinical trial, known as the PREHAB Trial [15], confirmed the results of earlier studies, including PHYSSURG-C [16] in which it is concluded that these programs based on exercise, nutrition, psychological support, and help to quit smoking when needed can reduce postoperative complications and enhance functional recovery.

The ERAS guidelines represent a paradigm shift in healthcare towards a patient-centered model. Incorporating patients’ perspectives during healthcare assessments is essential. The latest consensus recommendations emphasize the inclusion of quality-of-life parameters as perceived by patients, using patient-reported outcome measures (PROMs) and patient-reported experience measures (PREMs) to evaluate the quality of surgical interventions from the patient’s viewpoint [17,18]. PROMs focus on the patient’s self-reported health status and directly measure the impact of a disease or treatment from the patient’s perspective. PREMs reflect patients’ subjective experiences regarding the quality of care received and their interactions with the healthcare system and team throughout the care process. These parameters can engage the patient in their process, underscore their self-perception of their quality of life, and confirm the growing concern of health systems regarding patient satisfaction [19].

The digitalization of the healthcare process is essential for genuine and effective patient engagement. Digital systems enable continuous monitoring after discharge, integrate PROMs and PREMs into the clinical workflow, allow for personalized care, facilitate the early detection of problems, and enable population analysis to enhance services.

Healthcare technology is a reality, with an increasing number of mobile health applications being developed. However, many of these applications are created by non-medical companies. Despite this, the digitalization of society and the widespread accessibility of mobile devices capable of hosting these applications—over 80% in Europe and North America—support the adoption of these applications [20,21].

These applications can help systematize and personalize the information received by patients, improve follow-up, and enhance the overall patient experience. They have the potential to reduce anxiety and increase satisfaction with the care process [22]. Moreover, data recording and digitalization can lead to better adherence to medical recommendations [23] and improve patient monitoring. They also empower patients to take an active role in their recovery process and boost it, facilitating data-driven decision-making in the future.

The aim of the present study was to assess the capacity of a digital tool for remote monitoring of patients undergoing CRC surgery to optimize the care process by implementing an ERAS program. Additionally, we sought to examine clinical outcomes regarding perioperative morbidity and evaluate perceived quality in terms of value-based healthcare (VBHC) by objectively reporting results through PROMs and PREMs.

## 2. Materials and Methods

This study was conducted using a structured approach to integrate telemedicine into the patient’s healthcare journey and to facilitate safe communication between professionals and patients. Additionally, the VBHC model was followed, which focuses on understanding the efficiency of health outcomes by considering all dimensions of the healthcare process and including all professionals involved in the process in a transversal way [24].

The objective was to optimize the care process in patients with CRC by reducing inefficiencies in the process and implementing a digital solution for remote monitoring. This solution aims to improve the implementation and adherence to multimodal rehabilitation protocols, ultimately enhancing the efficiency of the process and the patient experience.

The methodology consisted of several phases. The first phase involved defining the study objectives and agreeing on the scope. Following this, a measurement phase was conducted to collect data for observational and process analyses. An analysis and evaluation of the results were performed, identifying areas for improvement and creating an action plan.

### 2.1. Analysis and Mapping of the Healthcare Process

A detailed analysis of patient care flow was conducted using the Value Stream Mapping (VSM) technique. This visual tool is used to analyze and improve processes by identifying each step of the workflow. In this case, the process began with the consultation providing surgery information and concluded with a follow-up consultation one month after hospital discharge. During this analysis, all workflow activities were identified, including contact points, waiting times, duplications, activities that do not add value, and the roles and responsibilities of each healthcare professional involved [25].

The analysis was based on the Lean Sigma process improvement methodology, which aims to minimize the time spent on non-value-adding activities, such as the variability within each task. The Lean Sigma methodology combines the removal of unnecessary elements within a process with the reduction in variability and errors to enhance overall process performance. The ultimate goal is to increase efficiency, quality, and patient satisfaction through continuous data-driven improvements [26].

### 2.2. Identification of Opportunities for Improvement

Based on the VSM analysis, the Kaizen principles of continuous improvement were applied within the organization. “Kaizen” means “change for the better” in Japanese, and it promotes that small, consistent changes over time can lead to significant improvements. As a result, specific areas of enhancement were identified that would directly impact efficiency and the patient experience. Issues such as patient information management, the implementation of multimodal prehabilitation programs, and improvement in outpatient care continuity were addressed [27].

### 2.3. Redesign and Digitalization of the Process

After identifying opportunities for improvement, the care process was redesigned to incorporate valuable elements. This new process was digitized into a digital solution for remote patient support, allowing for the implementation of ERAS guidelines. An internal ERAS protocol adapted to the recommendations of the ERAS Society was followed for all patients [28]. Additionally, a pentamodal prehabilitation program was designed and integrated into the digital solution, which includes: 1. Dietary recommendations; 2. A physical exercise plan tailored to the patient; 3. Emotional preparation and screening materials for anxiety and depression using the Hospital Anxiety and Depression Scale (HADS) for clinical psychology interventions if necessary; 4. An incentive respiratory exercise plan; and 5. The promotion of healthy lifestyle habits, including smoking cessation. The process was divided into four different stages for the patient: (a) a preparation protocol lasting 3 weeks, (b) a preoperative protocol during the 5 days leading up to surgery, (c) a protocol during the hospitalization period;, and (d) a final phase of 30 days after the patient’s discharge from the hospital.

Patients received daily recommendations tailored to their clinical characteristics (e.g., smoker, diabetic, alcohol consumer) and the stage of the care process they were undergoing (e.g., prehabilitation, immediate postoperative period). To assess adherence, they were sent a questionnaire every 24–48 h in which they reported whether they had followed the recommendations during that period.

### 2.4. Design and Validation of the Educational Program

An educational program with an individualized schedule was designed for the patients to ensure they received the necessary information in each part of the process and tailored to their specific profiles and conditions.

The clinical and educational content was created and validated by a multidisciplinary team that included surgeons, nurses, endocrinologists with expertise in nutrition, and psychologists. This collaboration ensured that the information was clinically appropriate, easy to understand for patients, and aligned with existing workflows.

### 2.5. Remote Monitoring

A protocol for remote patient monitoring was established, which included the development of automatic alert rules based on self-reported data and clinical variables. This approach enabled active and personalized monitoring, allowing for the early detection of clinical deviations, and facilitating proactive interventions by the care team.

Then, PROMs and PREMs were selected and integrated throughout the care process. These metrics assess the impact of the intervention from the patient’s perspective and are part of the VBHC assessment model.

### 2.6. Definition of Indicators, Dashboard of Indicators, and Outcome Measures

In the final phase of implementation, key impact indicators were established to evaluate the clinical, organizational, and experiential effectiveness of the digital solution. These indicators were selected based on their relevance to the VBHC model and included the following:

- Clinical outcomes (such as postoperative complications, length of hospital stay, mortality within 30 days).
- Indicators of adherence to the program.
- Usage levels of the digital solution.
- PROMs and PREMs collected at various stages of the process.
- Overall patient satisfaction.

These indicators were incorporated into a monitoring system using a digital dashboard, enabling real-time oversight, and promoting data-informed decision-making. This approach supports the continuous improvement of the care process through objective data and allows for monitoring of potential deviations from expected results.

### 2.7. Inclusion of Patients

Following the implementation of the solution, the patient inclusion phase was conducted. Patients meeting the following inclusion criteria were selected during the surgery indication consultation:

- Those who were aged eighteen years of age or older
- Those suitable for CRC surgery.
- Those with access to an email address, which is necessary for inclusion in the digital solution, either autonomously by the patient or with the assistance of a family member or caregiver who can help with the use and monitoring of the solution.
- Those with access to a smartphone, either autonomously by the patient or with the assistance of a family member or caregiver who can help with the use and monitoring of the solution.
- Those with the ability to follow the program through the digital solution, either autonomously by the patient or through the support of a family member or caregiver who can actively collaborate in the use and monitoring of the solution.

### 2.8. Exclusion of Patients

Patients who did not meet the inclusion criteria, and patients who were unable to follow the program through the digital solution either due to cognitive, physical, or technical limitations or without the necessary support from a family member or caregiver were excluded.

The study was approved by the Ethics Committee of the San Cecilio Clinical University Hospital. All participants received and signed an informed consent form prior to inclusion, authorizing their participation in the use of the application, clinical monitoring, and the collection and use of their data for research purposes. Patients received detailed information about the digital solution, including a description of the services, how personal data would be processed, and their rights. Once patients voluntarily and consciously signed the informed consent to participate, they were included in the digital solution by the healthcare team, and they began the monitoring program with the initial protocol for surgical preparation.

## 3. Results

From November 2023 to April 2025, a total of 227 patients were prospectively enrolled in the study, agreeing to use the digital solution. Out of these, 26 patients were excluded due to incomplete initial registration, and 8 unsubscribed from the digital solution (completed the registration but decided against it later).

Incomplete registration was defined by patients who initially agreed to use the digital solution and signed the informed consent form but who never completed the registration on the app, whereas unsubscribed patients completed the registration but withdrew from the app and the study before beginning to receive the prehabilitation questionnaires.

**Figure 1:**
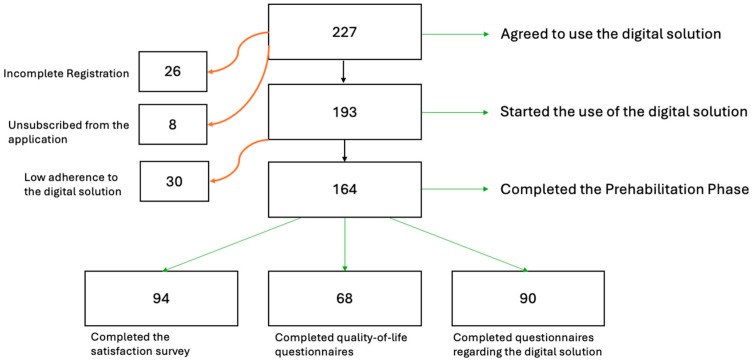
The flow of patients throughout the process.

In total, 193 patients initiated the use of the digital solution. Among them, 30 patients demonstrated low adherence, as they did not complete any of the inputs prompted by the app. Ultimately, 164 patients successfully completed the prehabilitation phase. At the end of the program, patients were asked to complete three questionnaires: a satisfaction survey, a quality-of-life questionnaire, and a global satisfaction questionnaire regarding the digital solution. The responses were collected from 94, 68, and 90 patients for each questionnaire, respectively (Figure 1).

### 3.1. Demographics

The mean age of participants was 67 years for men (N = 142, 62.5%) and 60 years for women (N = 85, 37.4%). The predominant age group for both sexes was between 50 and 75 years. In women, the most common diagnosis was rectal cancer (N = 21, 11%), while in men, the most frequent diagnoses were ascending colon cancer (N = 36, 18.75%), and rectal cancer (N = 35, 18.23%). The most common surgical procedures performed were a right hemicolectomy (N = 64, 33%), anterior rectal resection (N = 56, 29%), and a sigmoidectomy (N = 48, 25%). All surgeries were performed using either robotic or laparoscopic techniques, except for cases involving abdominoperineal resection and exploratory laparotomy.

Social risk screening using the Total Individual Risk Score (TIRS) scale identified 13 patients who were at risk, as assessed by the Social Work service. Additionally, the Fatigue, Resistance, Ambulation, Illness, Loss of Weight (FRAIL) scale identified 13 patients in a prefrailty state and 20 patients who were classified as fragile.

Table 1 describes the various diagnoses, procedures, and the main comorbidities, habits, and scales categorized by sex.

### 3.2. Adherence to the ERAS Protocol

**Figure 2:**
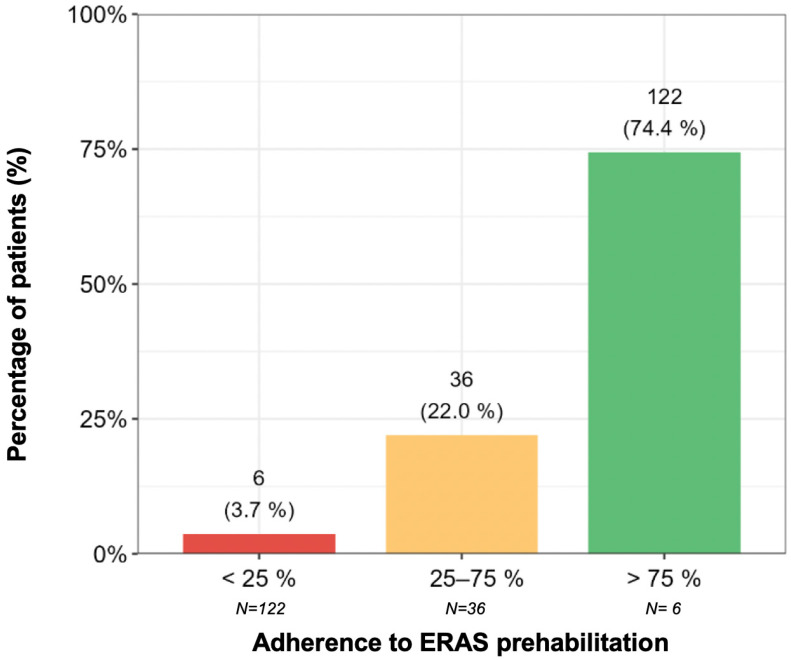
Distribution of the variable: adherence to the ERAS prehabilitation protocol.

Figure 2 shows the adherence to the ERAS recommendations categorized into three groups: high, intermediate, and low. Adherence was assessed based on the responses to sequential questionnaires delivered to patients via the app. Each day, patients received recommendations aligned with the multimodal ERAS protocol and were subsequently asked to report their compliance.

High adherence was found in 74.4% of patients (N = 122; 95% CI: 67.2–80.5%). Intermediate adherence was defined as compliance of between 25% and 75%, which was found in 22% of patients (N = 36; 95% CI: 16.3–28.9%). Low adherence, defined as compliance of less than 25%, was found in 3.7% of patients (N = 6; 95% CI: 1.7–7.8%).

### 3.3. Length of Hospital Stay (LoS)

The median LoS of the patients involved in the program was 5 days, with a range of 1 to 35 days (interquartile range (Q1–Q3): 4 to 8 days).

The relationship between adherence to ERAS recommendations and the LoS was analyzed, adjusting to a generalized linear model, assuming a negative binomial distribution of the median LoS variable.

**Figure 3:**
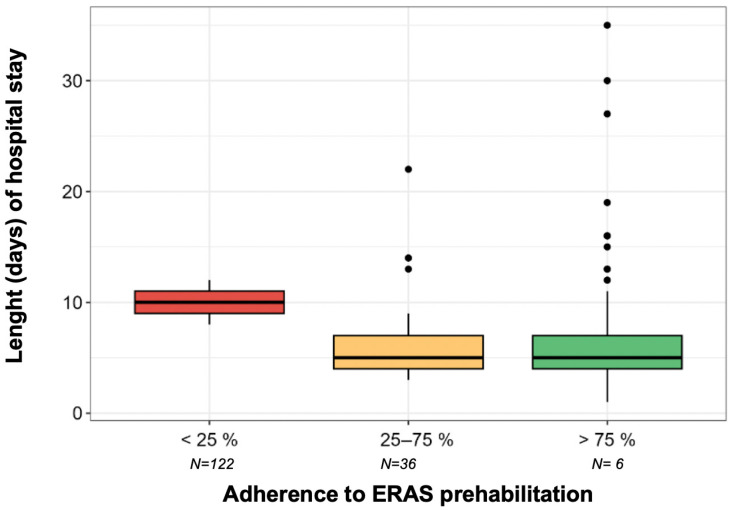
Forest Plot: relationship between adherence to ERAS and length (days) of hospital stay.

The findings indicated that patients with low adherence to the ERAS recommendations (<25%) had an increase in the median LoS of 3.4 days (*p* = 0.628) and 3.27 days (*p* = 0.642) compared to those in the intermediate- and high-adherence groups, respectively. However, these results were not statistically significant. To summarize, the median LoS for each adherence group was as follows:

- Low adherence (<25%): 10 days (95% CI: 4.45–22.48).
- Intermediate adherence (25–75%): 6.60 days (95% CI: 7.27–8.26).
- High adherence (>75%): 6.73 days (95% CI: 5.96–7.59) (Figure 3).

### 3.4. Comprehensive Complication Index (CCI)

Postoperative morbidity was assessed within the first 30 days using the comprehensive complication index (CCI), which reflects the accumulation of relevant complications in clinical progression based on the Clavien–Dindo classification and is weighted according to clinical significance [29].

The mean CCI for the patients included in the study was 9.1 per 100 patients (SD: 14.5).

There was one case of mortality within the first 30 days after surgery involving a 78-year-old woman. This was due to refractory septic shock following reoperation for ileocolic anastomosis dehiscence and aspiration pneumonia.

The relationship between postoperative morbidity and adherence to the ERAS recommendations was analyzed with a generalized linear model assuming a negative binomial distribution for the CCI variable.

The mean CCI was lower for the high-adherence group (7.42; 95% CI: 6.91–7.96) and the intermediate-adherence group (8.17; 95% CI: 7.21–9.26) than for the low-adherence group (15.00; 95% CI: 10.49–21.45).

**Figure 4:**
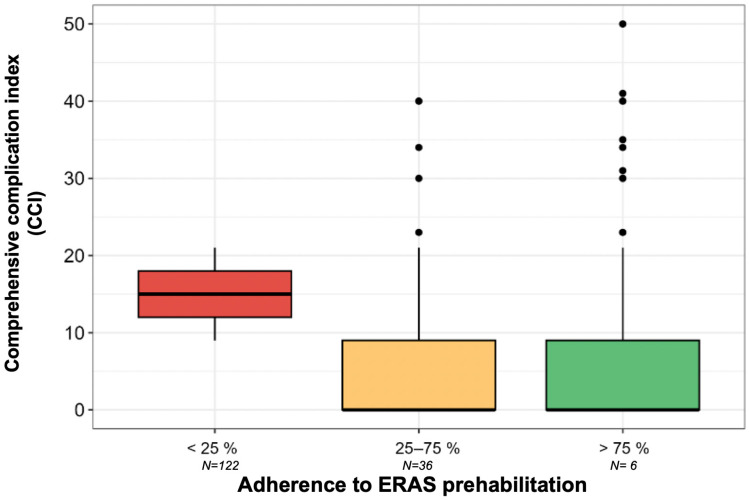
Forest Plot: relationship between postoperative morbidity at 30 days and adherence to ERAS protocol.

Compared to the low-adherence group, a decrease in the mean CCI of 6.83 (*p* = 0.027, 95%CI: −13.03 to −0.027) and 7.58 (*p* = 0.011, 95%CI: −13.7 to −1.47) was found for the intermediate- and high-adherence groups, respectively (Figure 4).

### 3.5. PROMs (Quality of Life)

A total of 68 patients completed the quality-of-life questionnaire based on the “Quality of Life Thermometer” of the validated EQ-5D scale [30]. The patients received the quality-of-life questionnaire at four different times: at the beginning of the program, postoperatively, two days after discharge, and 28 days after discharge.

**Figure 5:**
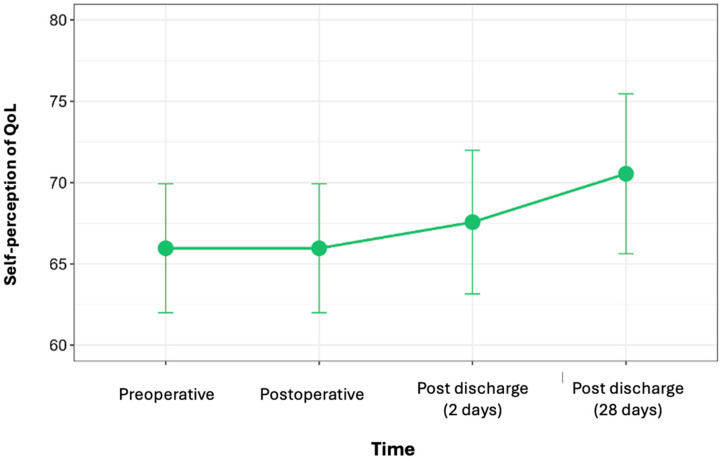
Changes in perception of quality of life (QoL) over time.

Figure 5 shows the variation in the self-perception of quality of life at different stages of the care process. An increase in the self-perception of quality of life by 9.2% was found at the end of the process compared to the outcome at the beginning of the process (*p* = 0.09; 95% CI: −0.622 to 9.788). We decided against performing a subgroup analysis due to the limited number of patients in the low-adherence group, which prevents effective segmentation of the variable across the different time points.

### 3.6. PREMs (Satisfaction and Experience)

At the end of the program, 94 patients completed the satisfaction survey. Among them, 80% rated their overall satisfaction with the healthcare process as 8 out of 10 or higher.

Patients rated the support they received during the care process, facilitated by the digital solution, with a median score of 8 out of 10. Additionally, 8 out of 10 patients felt that their disease management improved due to the digital solution. Furthermore, 9 out of 10 patients would recommend the digital solution, with 83% describing it as “very easy to use.”

## 4. Discussion

Despite advances in technology, the implementation of robotic and minimally invasive surgery, and the improvement in the support and treatment of patients with CRC, pose ongoing challenges for the scientific community in enhancing patient recovery. Complications can occur in over 50% of patients undergoing surgery, leading to a decline in their functional capacity and negatively impacting their life and individual health perceptions [31,32,33].

Effectively and sustainably implementing prehabilitation (ERAS) recommendations that appear to improve patients’ functional capacity, understanding, and patients’ perception, involving them in their healing process, and using innovative digital resources to address barriers to implementing these recommendations are important issues that should be integrated into surgical practice.

The application was used by 193 out of 227 patients who were included in the study and offered the opportunity to use it, demonstrating a wide acceptance among patients.

There is an increasing use of applications in healthcare that reflects the growing interest from the scientific community, strong patient acceptance, and the potential benefits that these technologies can offer in various phases of healthcare processes. In the surgical field, this trend is also evident, indicating that these solutions can complement traditional care and even improve health outcomes [34].

However, as this model of care evolves, it raises legal concerns regarding data protection and patient safety. Our solution complies with the National Security Scheme, which serves as the regulatory framework establishing the requirements and standards necessary to ensure the security of information in public administrations and their collaborating suppliers in Spain. The primary objective of this framework is to protect data and information systems by ensuring their confidentiality, integrity, and availability [35].

Most published studies on this topic only focus on postoperative recovery [36]. One novel aspect of the solution we present is its comprehensive monitoring of the patient throughout the entire surgical care process for CRC. This monitoring starts with the initial consultation providing information and continues postoperatively and after-discharge follow-up, concluding one month after the patient is discharged from the hospital.

ERAS recommendations in colorectal surgery indicate that various multimodal prehabilitation regimens can enhance patients’ functional capacity, decrease pre-operative anxiety, and encourage patients to take an active role in their health. These factors improve patient recovery, as demonstrated by objective data showing a decrease in complications and a shorter LoS [14,15].

Most of these programs are derived from interventions within research projects that engage professionals from various fields (such as trainers and physical exercise experts), which may not be sustainable over time, at least in the short term, due to the need for additional personnel resources. Additionally, some initiatives rely on “homemade” recommendation sheets without the ability to monitor patient compliance effectively. These challenges may explain the inconsistent results observed in other studies, where the true impact of prehabilitation remains unclear, likely due to variability and difficulties in its implementation [37,38].

We can assert that the digital solution plays a critical role in monitoring and optimizing the care process of patients with CRC, accommodating the unique characteristics and needs of each medical center. These solutions also facilitate realistic and sustainable participation from professionals across various specialties, including physical activity experts, rehabilitators, nutritionists, psychologists, oncologists, gastroenterologists, and anesthesiologists. All recommendations, information, and questionnaires were delivered sequentially to the patient, which enhances adherence to these recommendations and allows for more objective daily tracking of compliance. In addition to improving adherence, this approach enhances patient involvement, as our results demonstrate.

Based on our experience, implementing the digital solution can effectively facilitate the adoption of ERAS measures throughout the CRC process. Our adherence results indicate that a substantial majority of patients (>95%) were able to easily and sequentially complete most of the prehabilitation recommendations (>75%) from home, which is likely to benefit their recovery and reduce their LoS and complication rates.

The results of the present study regarding key variables related to patients’ functional recovery, such as the CCI score and LoS, are consistent with findings published by leading research groups and early meta analyses. For instance, in the randomized PREHAB TRIAL, Molenaar et al. found a reduction in postoperative medical complications in the prehabilitation group (odds ratio, OR = 0.48) and a faster and better recovery of functional capacity compared to the standard care group. Additionally, Forsmo et al. found a significant decrease in the median LoS, from 8 days to 5 days, which is consistent with our findings; however, they found no significant differences in postoperative complications [15,39,40,41].

Furthermore, the flexibility of the application would allow for the development of programs tailored to each patient’s profile. One important consideration in implementing ERAS prehabilitation programs is the potential for enhanced benefits for patients who require postoperative cancer therapies, including chemotherapy or immunotherapy, or who may need subsequent surgeries for metastases or carcinomatosis [42,43,44].

In our series, patient adherence to the ERAS recommendations was notably high, with 74.4% of patients following more than 75% of the ERAS recommendations. The application has received positive feedback: 8 out of 10 patients feel their disease management has improved with this application, and 9 out of 10 would recommend it, as it helps them maintain a sense of follow-up before and after surgery. This application modernizes and enhances the care process, which may contribute to tangible improvements in patients’ recovery, as evidenced by reductions in the LoS and the CCI. It facilitates the effective implementation and adherence to ERAS recommendations, during the preoperative (prehabilitation) and postoperative phases.

Current trends in healthcare quality management strongly influence the concept of Value-Based Health Care (VBHC), introduced by Porter in 2008 [45], which relates health outcomes to the costs required to achieve them. Proper analysis requires evaluating outcomes across all phases of care and involving all professional stakeholders [46,47]. In this context, patient-reported measures such as PREMs and PROMs are essential but often underutilized. Outcome assessments frequently focus only on limited aspects of care, like postoperative indicators (e.g., surgical site infections, length of stay), which, while relevant, offer an incomplete view. Similarly, costs are often measured by volume (e.g., number of CRC surgeries) rather than quality or patient impact (e.g., reoperations or readmissions). As our study suggests, investing in specific stages—such as prehabilitation—can reduce downstream costs, including through shorter hospital stays and fewer complications.

Despite inherent observational limitations, we found a decrease in the median LoS in patients with a higher adherence to the prehabilitation process. Those with adherence rates of between 25% and 100% had a median LoS below 7 days, while those who did not follow the prehabilitation process had a median LoS of 10 days. Given the preliminary nature of this study and the small sample size within adherence subgroups, no covariate adjustment was performed in the current models. We acknowledge that this may introduce potential confounding effects. It is also important to note that the low-adherence group included only six patients, which substantially limits the statistical power to detect significant differences, increases the risk of type II error, and may obscure potentially meaningful associations.

However, despite these constraints, the observed trends remain encouraging and suggest potential clinical relevance. We plan to overcome these shortcomings with more robust multivariable analyses in future studies involving larger cohorts.

Effective VBHC analysis requires two core approaches: a longitudinal view of the care process and integration of the patient perspective via PROMs and PREMs. A digital solution could support both more efficiently.

Few studies have systematically captured the patient perspective in the CRC pathway [48], but standardizing PROM and PREM data collection could improve care quality and provide a reliable indicator of overall performance. Our study shows high patient satisfaction with the digital solution (8 out of 10). They perceived the support they received during the care process, facilitated by the digital solution, with a median score of 8 out of 10. These results confirm that digital solutions such as the one discussed in the present study can improve the value of our actions from the patient’s perspective.

Furthermore, in analyzing the perceived quality of life, we found a gradual increase at the four time points when the questionnaires were administered (Figure 5). Unfortunately, the small sample size in the subgroups with lower adherence to recommendations has limited our ability to conduct a subgroup analysis based on adherence; however, it would be interesting for further studies.

Our study aims to present preliminary, descriptive results, and we recognize the limitations of drawing evidence-based conclusions. Firstly, the relationship between clinical data—specifically recovery, the LoS, and complications (CCI)— may be biased due to the impact of multimodal prehabilitation measures alone. Therefore, we need to continue our research by conducting prospective studies randomized with a control group. This will allow us to measure not only adherence to the prehabilitation measures but also the actual impact on complications and recovery. It will also help to clarify the role that these digital solutions play when compared to “usual care”, where such solutions are absent.

Another potential limitation of this study is self-selection bias. Patients who adhered to the treatment may differ systematically from those who did not, in ways that could influence outcomes independently of the intervention (e.g., motivation, education, health awareness). Moreover, the small number of non-adherent patients limits the reliability of between-group comparisons and the generalizability of the results. However, given the preliminary nature of these findings, a highly motivated and homogeneous sample was expected at this early stage. In future analyses, involving larger and more diverse cohorts, we will conduct a more comprehensive assessment of potential self-selection biases to better account for their impact and to enable a more robust evaluation of adherence effects.

While our data on adherence appear positive, we acknowledge that these findings are relative due to the lack of a standard for comparison. We believe that systematizing the use of the digital solution and redesigning the patient flow within a study that includes a control group would help to minimize subjects lost to follow-up and lead to more reliable conclusions.

The digital solution simplifies the registration of PROMS and PREMS data through an electronic format known as “ePROMs and ePREMS”. However, no agreement has been reached on which validated questionnaires best capture these concepts, leading to a lack of standardization [49].

Moreover, many healthcare professionals are unfamiliar with these concepts and may question their validity or significance. There is a perception among some that these measures may be redundant in a patient’s medical history or lack a genuine impact on health outcomes.

In light of the considerations previously outlined and the results obtained, we propose the following improvements for future studies:

1. A randomized controlled trial (RCT) will be designed, including a control group that receives standard care and access to ERAS recommendations in a paper format, but without exposure to the digital solution. This will allow us to assess the comparative impact of the app-based intervention on all aspects of perioperative care in patients with colorectal cancer. This study design will directly address key methodological limitations of the current study, including the absence of a control group, limited standardization of outcome measures, and potential selection bias.

2. A larger study population will be included to increase the statistical power and enable more comprehensive multivariable analyses, accounting for potential confounding factors. Furthermore, potential sources of selection bias will be systematically explored and addressed to enhance the robustness of the findings.

3. In addition, validated and widely accepted tools for assessing patient-reported outcomes and experiences will be used, such as the EQ-5D-5L, which offers standardized assessment of health-related quality of life and facilitates comparison across studies and settings.

Our digital solution enhances active patient participation in their own healthcare and captures objective data about their perspectives and experiences across various areas. The data collected, along with our experience with the digital solution, lead us to believe we can effectively register these variables and identify which ones best represent the patient’s viewpoint. Additionally, this process promotes a positive culture for the use of this digital solution within the medical profession. These well-analyzed data can provide powerful insights to modify, reinforce, or change specific aspects of healthcare processes. This will enable us to reproduce them and implement truly patient-centered and value-based healthcare.

## 5. Conclusions

Digital solutions such as the one we propose in the present study can facilitate the review of the CRC care process and support the implementation and adherence to ERAS recommendations in multimodal prehabilitation (and therefore, enhance their benefits in terms of improving the functional capacity and recovery of patients). This solution can also help to engage patients in their recovery process, and record and objectively improve their perceptions through PREMS and PROMS, all in line with the principles of VBHC. However, further prospective studies are needed to clearly define the actual value of this intervention compared to a control group.

## Figures and Tables

**Table 1:** Demographic, epidemiological, and clinical characteristics of the sample (BMI: Body Mass Index).

	Men		Women	
	N	%	N	%
Demographics				
N	142	62.56%	85	37.44%
Age (mean) years	67		70	
<50	9	3.96%	5	2.20%
50–75	95	41.85%	42	18.50%
>75	38	16.74%	38	16.74%
Diagnosis				
Right colon cancer	36	18.75%	19	9.90%
Rectal cancer	35	18.23%	21	10.94%
Sigmoid colon cancer	33	17.19%	17	8.85%
Left colon cancer	12	6.25%	10	5.21%
Cecum cancer	2	1.04%	4	2.08%
Transverse colon cancer	3	1.56%	-	-
Procedure				
Right hemicolectomy	41	20.60%	23	11.56%
Sigmoidectomy	33	16.58%	15	7.54%
Anterior rectal resection	25	12.56%	20	10.05%
Left hemicolectomy	16	8.04%	8	4.02%
Local resection	3	1.51%	5	2.51%
Abdominoperineal resection	3	1.51%	2	1.01%
Exploratory Laparotomy	1	0.50%	1	0.50%
Pancolectomy	1	0.50%	1	0.50%
Subtotal colectomy	1	0.50%	-	-
Medical history and risk factors				
Smoker	12	6.86%	5	2.86%
Former smoker	54	30.86%	21	12%
Alcohol consumption (>14 consumptions/week)	49	28%	13	7.43%
Diabetes	19	11.66%	10	6.13%
Hypertension	49	30.06%	31	19.02%
Overweight (BMI: 25–30)	47	26.55%	28	15.82%
Obesity (BMI: >30)	34	19.21%	14	7.91%
Cardiovascular disease				
Heart disease	20	12.27%	9	5.52%
Arrhythmia	11	37.93%	2	6.9%
Myocardial ischemia history	7	24.14%	2	6.9%
Chronic kidney disease	12	7.36%	6	3.68%
Lung disease	11	6.75%	11	6.75%

## Data Availability

The data and material used and analyzed in the current study are available from the corresponding author.

## Abbreviations

The following abbreviations are used in this manuscript:

CCI	Comprehensive Complication Index
CRC	Colorectal cancer
ERAS	Enhanced Recovery After Surgery
FRAIL	Fatigue, Resistance, Ambulation, Illness, Loss of Weight
HADS	Hospital Anxiety and Depression Scale
LoS	Length of hospital stay
PROM	Patient-reported outcome
PREM	Patient-reported experience
TIRS	Total Individual Risk Score
VBHC	Value-based healthcare
VSM	Value Stream Mapping

## References

[1] Perera S.K., Jacob S., Wilson E.B., Ferlay J., Bray F., Sullivan R., Barton M. (2021). Global demand for cancer surgery and an estimate of the optimal surgical and anaesthesia workforce between 2018 and 2040: A population-based modelling study. Lancet Oncol..

[2] Lawrence V.A., Hazuda H.P., Cornell J.E., Pederson T., Bradshaw P.T., Mulrow C.D., Page C.P. (2004). Functional independence after major abdominal surgery in the elderly. J. Am. Coll. Surg..

[3] Stabenau H.F., Becher R.D., Gahbauer E.A., Leo-Summers L., Allore H.G., Gill T.M. (2018). Functional Trajectories Before and After Major Surgery in Older Adults. Ann. Surg..

[4] Sharp S.P., Malizia R., Skancke M., Arsoniadis E.G., Ata A., Stain S.C., Valerian B.T., Lee E.C., Wexner S.D. (2020). A NSQIP analysis of trends in surgical outcomes for rectal cancer: What can we improve upon?. Am. J. Surg..

[5] Steffens D., Ismail H., Denehy L., Beckenkamp P.R., Solomon M., Koh C., Bartyn J., Pillinger N. (2021). Preoperative Cardiopulmonary Exercise Test Associated with Postoperative Outcomes in Patients Undergoing Cancer Surgery: A Systematic Review and Meta-Analyses. Ann. Surg. Oncol..

[6] Wischmeyer P.E., Carli F., Evans D.C., Guilbert S., Kozar R., Pryor A., Thiele R.H., Everett S., Grocott M., Gan T.J. (2018). American Society for Enhanced Recovery and Perioperative Quality Initiative Joint Consensus Statement on Nutrition Screening and Therapy within a Surgical Enhanced Recovery Pathway. Anesth. Analg..

[7] Molenaar C.J.L., Janssen L., van der Peet D.L., Winter D.C., Roumen R.M.H., Slooter G.D. (2021). Conflicting Guidelines: A Systematic Review on the Proper Interval for Colorectal Cancer Treatment. World J Surg..

[8] Barberan-Garcia A., Ubré M., Roca J., Lacy A.M., Burgos F., Risco R., Momblán D., Balust J., Blanco I., Martínez-Pallí G. (2018). Personalised prehabilitation in high-risk patients undergoing elective major abdominal surgery: A randomized blinded controlled trial. Ann. Surg..

[9] Barker A.L., Gray C., Wilson L., Thomson B.N.J., Shedda S., Crowe T.C. (2013). Preoperative immunonutrition and its effect on postoperative outcomes in well-nourished and malnourished gastrointestinal surgery patients: A randomised controlled trial. Eur. J. Clin. Nutr..

[10] Bousquet-Dion G., Awasthi R., Loiselle S., Minnella E.M., Agnihotram R.V., Bergdahl A., Carli F., Scheede-Bergdahl C. (2018). Evaluation of supervised multimodal prehabilitation programme in cancer patients undergoing colorectal resection: A randomized control trial. Acta Oncol..

[11] Burden S.T., Gibson D.J., Lal S., Hill J., Pilling M., Soop M., Ramesh A., Todd C. (2017). Pre-operative oral nutritional supplementation with dietary advice versus dietary advice alone in weight-losing patients with colorectal cancer: Single-blind randomized controlled trial. J. Cachexia Sarcopenia Muscle.

[12] Minnella E.M., Ferreira V., Awasthi R., Charlebois P., Stein B., Liberman A.S., Scheede-Bergdahl C., Morais J.A., Carli F. (2020). Effect of two different pre-operative exercise training regimens before colorectal surgery on functional capacity. Eur. J. Anaesthesiol..

[13] Polakowski C.B., Kato M., Preti V.B., Schieferdecker M.E.M., Campos A.C.L. (2019). Impact of the preoperative use of synbiotics in colorectal cancer patients: A prospective, randomized, double-blind, placebo-controlled study. Nutrition.

[14] Onerup A., Andersson J., Angenete E., Bock D., Börjesson M., Ehrencrona C., Olsén M.F., Larsson P.-A., de la Croix H., Wedin A. (2021). Effect of Short-term Homebased Pre- and Postoperative Exercise on Recovery After Colorectal Cancer Surgery (PHYSSURG-C). Ann. Surg..

[15] Molenaar C.J.L., Minnella E.M., Coca-Martinez M., Cate D.W.G.T., Regis M., Awasthi R., Martínez-Palli G., López-Baamonde M., Sebio-Garcia R., Feo C.V. (2023). Effect of Multimodal Prehabilitation on Reducing Postoperative Complications and Enhancing Functional Capacity Following Colorectal Cancer Surgery. JAMA Surg..

[16] van Rooijen S., Carli F., Dalton S., Thomas G., Bojesen R., Le Guen M., Barizien N., Awasthi R., Minnella E., Beijer S. (2019). Multimodal prehabilitation in colorectal cancer patients to improve functional capacity and reduce postoperative complications: The first international randomized controlled trial for multimodal prehabilitation. BMC Cancer.

[17] Domenghino A., Walbert C., Birrer D.L., Puhan M.A., Clavien P.-A. (2023). Consensus recommendations on how to assess the quality of surgical interventions. Nat. Med..

[18] Fiore J.F., Figueiredo S., Balvardi S., Lee L., Nauche B., Landry T., Mayo N.E., Feldman L.S. (2018). How Do We Value Postoperative Recovery?. Ann. Surg..

[19] Burch J., Taylor C. (2012). Patients’ need for nursing telephone follow-up after enhanced recovery. Gastrointest. Nurs..

[20] Anderson M. (2019). Mobile Technology and Home Broadband 2019.

[21] Krebs P., Duncan D.T. (2015). Health App Use Among US Mobile Phone Owners: A National Survey. JMIR mHealth uHealth.

[22] Kumar D., Arya M. (2015). mHealth is an Innovative Approach to Address Health Literacy and Improve Patient-Physician Communication—An HIV Testing Exemplar. J. Mob. Technol. Med..

[23] Eustache J., El-Kefraoui C., Ekmekjian T., Latimer E., Lee L. (2021). Do postoperative telemedicine interventions with a communication feature reduce emergency department visits and readmissions?—A systematic review and meta-analysis. Surg. Endosc..

[24] van Staalduinen D.J., van den Bekerom P., Groeneveld S., Kidanemariam M., Stiggelbout A.M., van den Akker-van Marle M.E. (2022). The implementation of value-based healthcare: A scoping review. BMC Health Serv. Res..

[25] Marin-Garcia J.A., Vidal-Carreras P.I., Garcia-Sabater J.J. (2021). The Role of Value Stream Mapping in Healthcare Services: A Scoping Review. Int. J. Environ. Res. Public Health.

[26] Mason S., Nicolay C., Darzi A. (2015). The use of Lean and Six Sigma methodologies in surgery: A systematic review. Surgeon.

[27] Goyal S., Law E. (2019). An introduction to Kaizen in health care. Br. J. Hosp. Med..

[28] Gustafsson U., Scott M., Schwenk W., Demartines N., Roulin D., Francis N., McNaught C., MacFie J., Liberman A., Soop M. (2012). Guidelines for perioperative care in elective colonic surgery: Enhanced Recovery After Surgery (ERAS^®^) Society recommendations. Clin. Nutr..

[29] Slankamenac K., Nederlof N., Pessaux P., de Jonge J., Wijnhoven B.P.L., Breitenstein S., Oberkofler C.E., Graf R., Puhan M.A., Clavien P.-A. (2014). The Comprehensive Complication Index. Ann. Surg..

[30] The EuroQol Group (1990). EuroQol-a new facility for the measurement of health-related quality of life. Health Policy.

[31] Kirchhoff P., Clavien P.-A., Hahnloser D. (2010). Complications in colorectal surgery: Risk factors and preventive strategies. Patient Saf. Surg..

[32] McDermott F.D., Heeney A., Kelly E.M., Steele R.J., Carlson G.L., Winter D.C. (2015). Systematic review of preoperative, intraoperative and postoperative risk factors for colorectal anastomotic leaks. Br. J. Surg..

[33] Govaert J., Fiocco M., van Dijk W., Scheffer A., de Graaf E., Tollenaar R., Wouters M., Lamme B., Hess D., Belgers H. (2015). Costs of complications after colorectal cancer surgery in the Netherlands: Building the business case for hospitals. Eur. J. Surg. Oncol..

[34] Popa V., Geissler J., Vermeulen R., Priest E., Capperella K., Susuzlu G., Terry S.F., Brooke N. (2023). Delivering Digital Health Solutions that Patients Need: A Call to Action. Ther. Innov. Regul. Sci..

[35] Boletín Oficial del Estado (2022). Real Decreto 311/2022, de 3 de mayo, por el que se Regula el Esquema Nacional de Seguridad.

[36] He M., Chen M., Ji Y., Lu G. (2024). Effectiveness of smartphone app-based interventions after surgery on quality of recovery among cancer patients: A systematic review and meta-analysis. Ann. Med..

[37] Carli F., Bousquet-Dion G., Awasthi R., Elsherbini N., Liberman S., Boutros M., Stein B., Charlebois P., Ghitulescu G., Morin N. (2020). Effect of Multimodal Prehabilitation vs Postoperative Rehabilitation on 30-Day Postoperative Complications for Frail Patients Undergoing Resection of Colorectal Cancer. JAMA Surg..

[38] Gloor S., Misirlic M., Frei-Lanter C., Herzog P., Müller P., Schäfli-Thurnherr J., Lamdark T., Schregel D., Wyss R., Unger I. (2022). Prehabilitation in patients undergoing colorectal surgery fails to confer reduction in overall morbidity: Results of a single-center, blinded, randomized controlled trial. Langenbeck’s Arch. Surg..

[39] Forsmo H.M., Pfeffer F., Rasdal A., Østgaard G., Mohn A.C., Körner H., Erichsen C. (2016). Compliance with enhanced recovery after surgery criteria and preoperative and postoperative counselling reduces length of hospital stay in colorectal surgery: Results of a randomized controlled trial. Color. Dis..

[40] Lv L., Shao Y.-F., Zhou Y.-B. (2012). The enhanced recovery after surgery (ERAS) pathway for patients undergoing colorectal surgery: An update of meta-analysis of randomized controlled trials. Int. J. Color. Dis..

[41] Berian J.R., Ko C.Y., Ban K.A. (2019). Does Implementation of Enhanced Recovery after Surgery (ERAS) Protocols in Colorectal Surgery Improve Patient Outcomes?. Clin. Colon Rectal Surg..

[42] van Waart H., Stuiver M.M., van Harten W.H., Sonke G.S., Aaronson N.K. (2010). Design of the Physical exercise during Adjuvant Chemotherapy Effectiveness Study (PACES): A randomized controlled trial to evaluate effectiveness and cost-effectiveness of physical exercise in improving physical fitness and reducing fatigue. BMC Cancer.

[43] van Rooijen S.J., Engelen M.A., Scheede-Bergdahl C., Carli F., Roumen R.M.H., Slooter G.D., Schep G. (2017). Systematic review of exercise training in colorectal cancer patients during treatment. Scand. J. Med. Sci. Sports.

[44] De Backer I.C., Van Breda E., Vreugdenhil A., Nijziel M.R., Kester A.D., Schep G. (2007). High-intensity strength training improves quality of life in cancer survivors. Acta Oncol..

[45] Porter M.E. (2008). Value-Based Health Care Delivery. Ann. Surg..

[46] Feldman L.S., Lee L., Fiore J. (2014). What outcomes are important in the assessment of Enhanced Recovery After Surgery (ERAS) pathways?. Can. J. Anaesth..

[47] Krogsgaard M.R., Brodersen J., Christensen K.B., Siersma V., Kreiner S., Jensen J., Hansen C.F., Comins J.D. (2020). What is a PROM and why do we need it?. Scand. J. Med. Sci. Sports.

[48] Lau J., Ng J.S., Lee D., Tan J.K., Tan L.L.Y., Pang N.Q., Tham S.Y., Ng C.K., Tan K.K. (2024). Use of patient-reported experience and outcome measures within the colorectal cancer care continuum: A scoping review. J. Cancer Surviv..

[49] Iroz C.B., Johnson J.K., Ager M.S., Joung R.H.-S., Brajcich B.C., Cella D., Franklin P.D., Holl J.L., Bilimoria K.Y., Merkow R.P. (2023). Barriers and Facilitators to Implementing Patient-Reported Outcome Monitoring in Gastrointestinal Surgery. J. Surg. Res..

